# Supplementary material for: The Effect of Alkyl Substituents on the Formation and Structure of Homochiral (R*,R*)-[R2Ga(µ-OCH(Me)CO2R′)]2 Species—Towards the Factors Controlling the Stereoselectivity of Dialkylgallium Alkoxides in the Ring-Opening Polymerization of rac-Lactide
Source: Molecules. 2025 Jan 6;30(1):190. doi: 10.3390/molecules30010190 (PMC11721103; doi:10.3390/molecules30010190)
Supplement: Supplementary file 1 [file molecules-30-00190-s001.zip › molecules-3393806-supplementary.pdf]

Supplementary Materials

The Effect of Alkyl Substituents on the Formation and Structure of Homochiral (*R**,*R**)-[R2Ga(*μ*-OCH(Me)CO2R')]2 Species—Towards the Factors Controlling the Stereoselectivity of Dialkylgallium Alkoxides in the Ring-Opening Polymerization of *rac*-Lactide

Magdalena Kaźmierczak [1,2], Łukasz Dobrzycki [3], Maciej Dranka [1], and Paweł Horeglad [1,*]

[1] Faculty of Chemistry, Warsaw University of Technology, Noakowskiego 3, 00-664, Warsaw, Poland; kazmierczak.magdalena.m@gmail.com (M.K.); maciej.dranka@pw.edu.pl (M.D.)

[2] *Centre of New Technologies, University of Warsaw, Banacha 2c, 02-097, Warsaw, Poland.*

[3] *Faculty of Chemistry, University of Warsaw, Pasteura 1, 02-093, Warsaw, Poland; lm.dobrzycki@gmail.com*

* *Correspondence: pawel.horeglad@pw.edu.pl; Tel.: +48-22-234-5076*

1) **Selected ^{1}H NMR data for gallium complexes (Figures S1 – S7)**
2) **Exemplary ^{1}H NMR spectra of PLA (Figure S8)**
3) **Decoupled ^{1}H NMR spectra of PLA obtained with (*S,S*)-[R$_2$Ga(*μ*-OCH(Me)CO$_2$Me]$_2$ (Figures S9 – S11)**
4) **FTIR data for (*S,S*)-1 and (*S,S*)-2 (Figures S12 – S13)**
5) **MALDI-TOF of PLA obtained with complexes 1 and 3 (Figures S14 – S16)**
6) **Crystallographic data of** (*R,S*)-**2**, (*S,S*)-**1**, (*S,S*)-**2**, (*R,S*)-**3** and (*R,S*)-**4.**

1) ^{1}H NMR Data

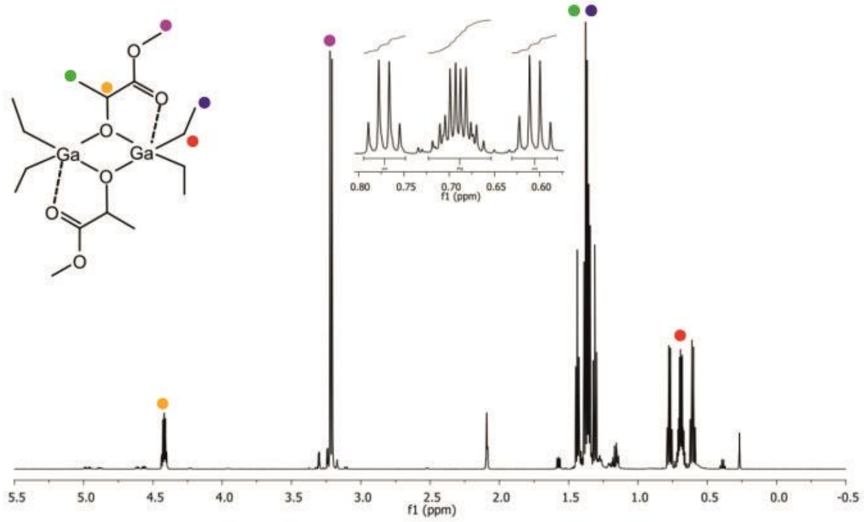

Figure S1. ^{1}H NMR (toluene-d_8, 700 MHz) spectrum of **1**.

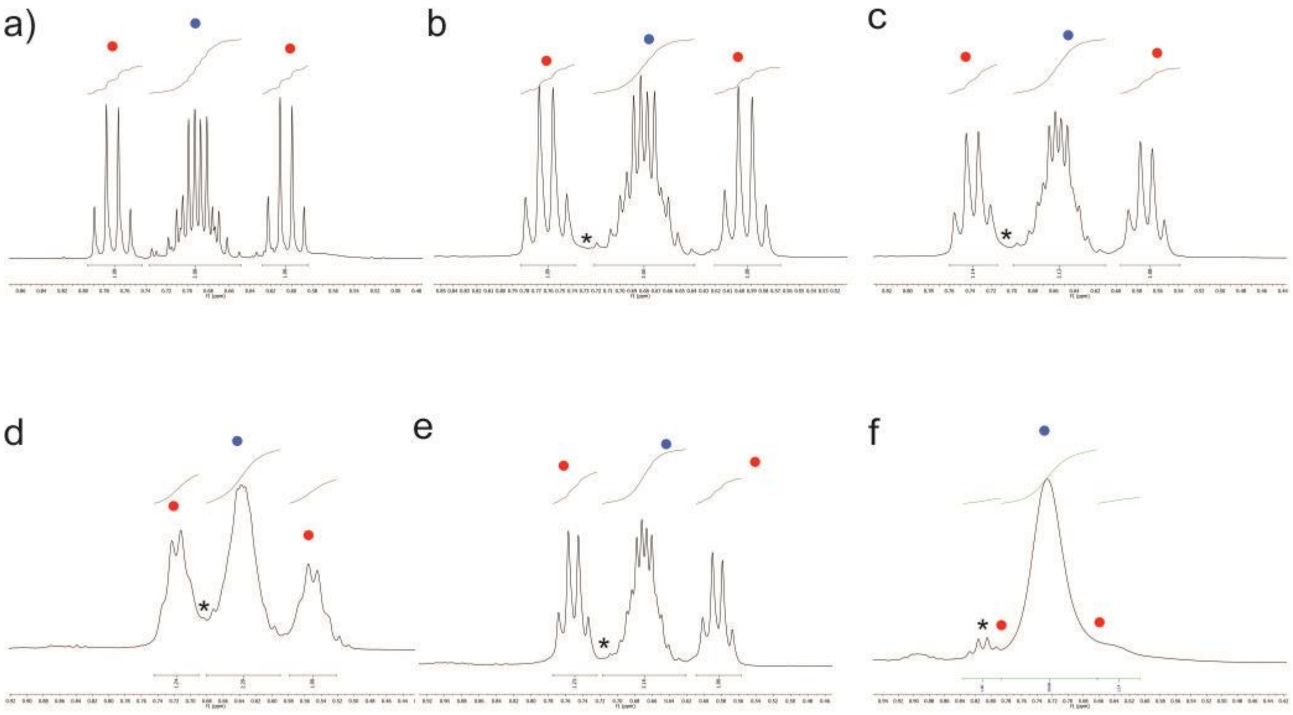

Figure S2. ^{1}H NMR spectra of **1** and **1**/*Py* in the region corresponding to methylene protons of Ga–CH_2CH_3 groups - (**1** (a), **1**/pyridine (1:2) (b), **1**/pyridine (1:6) (c), **1**/pyridine (1:60) (d), **1**/ 4-methylpyridine (1:6) (e), **2**/4-(dimethylamino)pyridine (1:6) (f)). (*R*,R**)-**1** (•) and (R,S)-**1** (•) are depicted as blue and red dots, respectively. Tentative monomeric $Et_2Ga(OCH(Me)CO_2Me)$(*Py*) species are depicted as stars (∗); The integral corresponding to the latter was estimated based on the fact that both septets corresponding to *R,S*-**2** refer to the same number of methine protons. Therefore

the integral of the signal at around 0.77 ppm, which could be affected by the presence of a tentative monomeric species was assumed to be equal to the one for the signal at around 0.66 ppm.

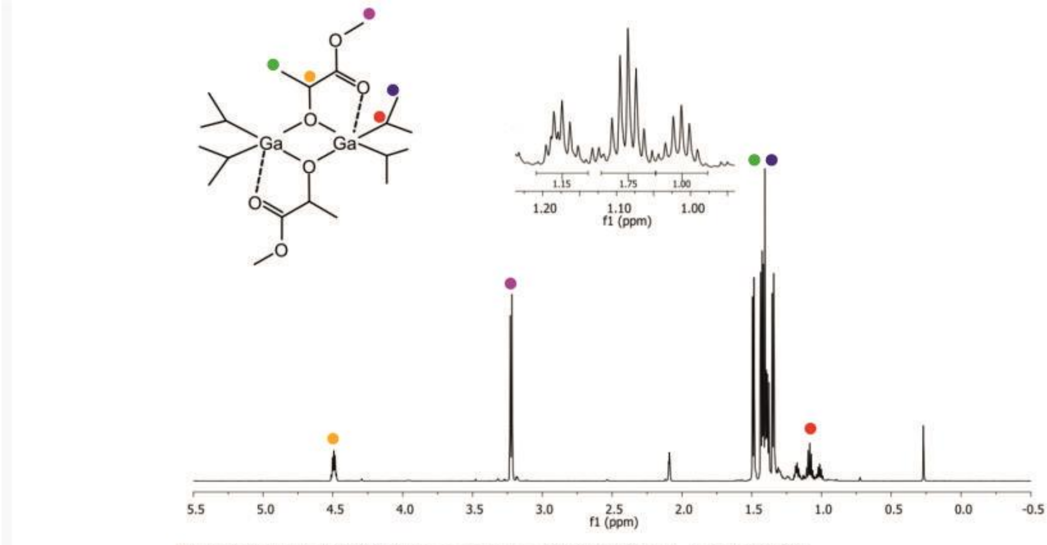

Rysunek 19 Widmo ^{1}HNMR związku rac-[^{i}Pr$_2$Ga(μ-OCH(Me)CO$_2$Me)]$_2$ (toluen, 700MHz).

Figure S3. ^{1}H NMR (toluene-d_8, 700 MHz) spectrum of **2**.

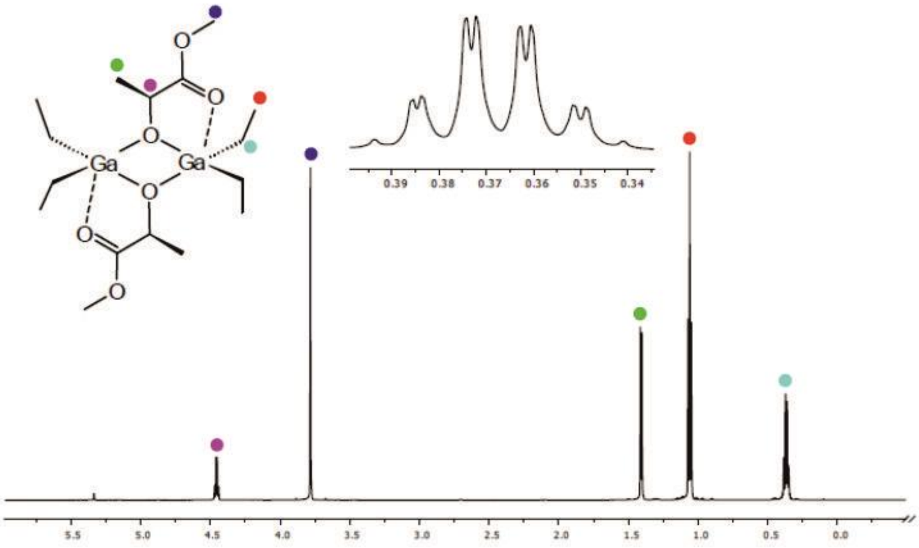

Figure S4. ^{1}H NMR (CD_2Cl_2, 700 MHz) spectrum of (*S*,*S*)-**1**.

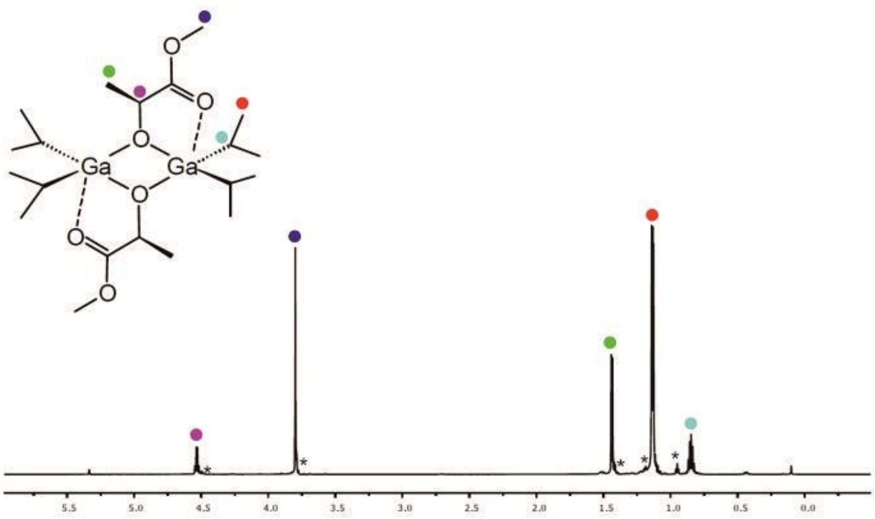

Figure S5. ^{1}H NMR (CD_2Cl_2, 700 MHz) spectrum of (*S*,*S*)-**2**. Tentative monomeric species are indicated with *

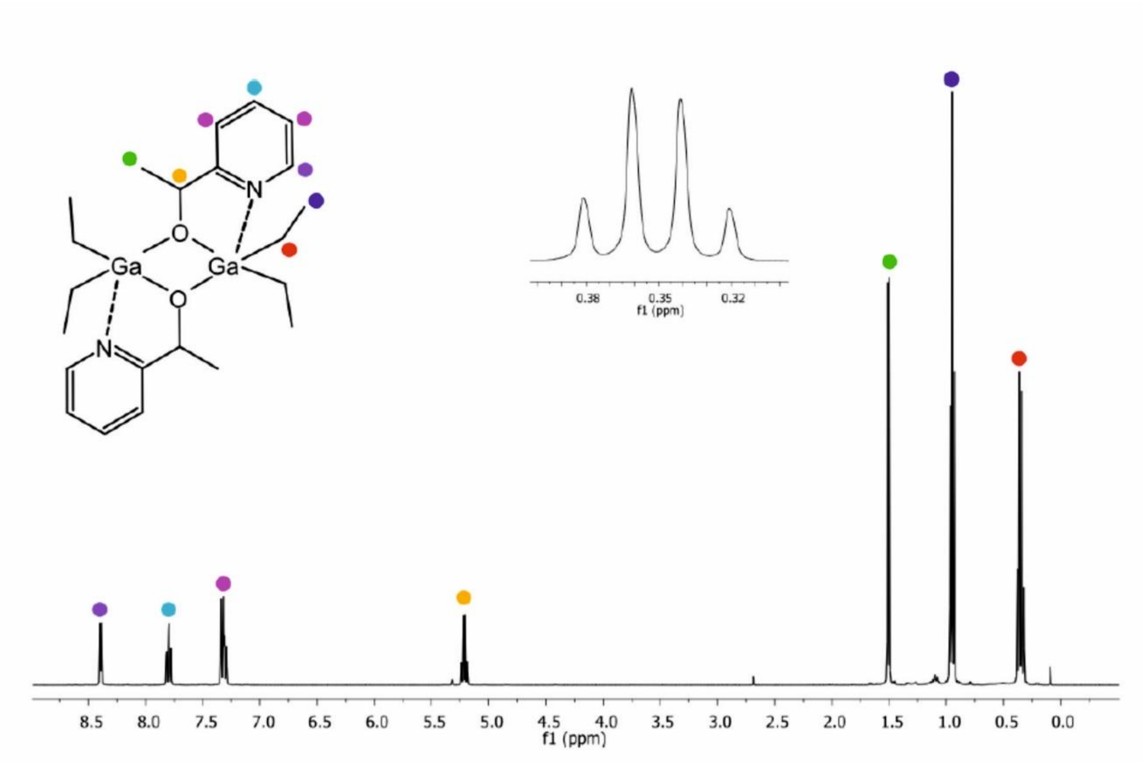

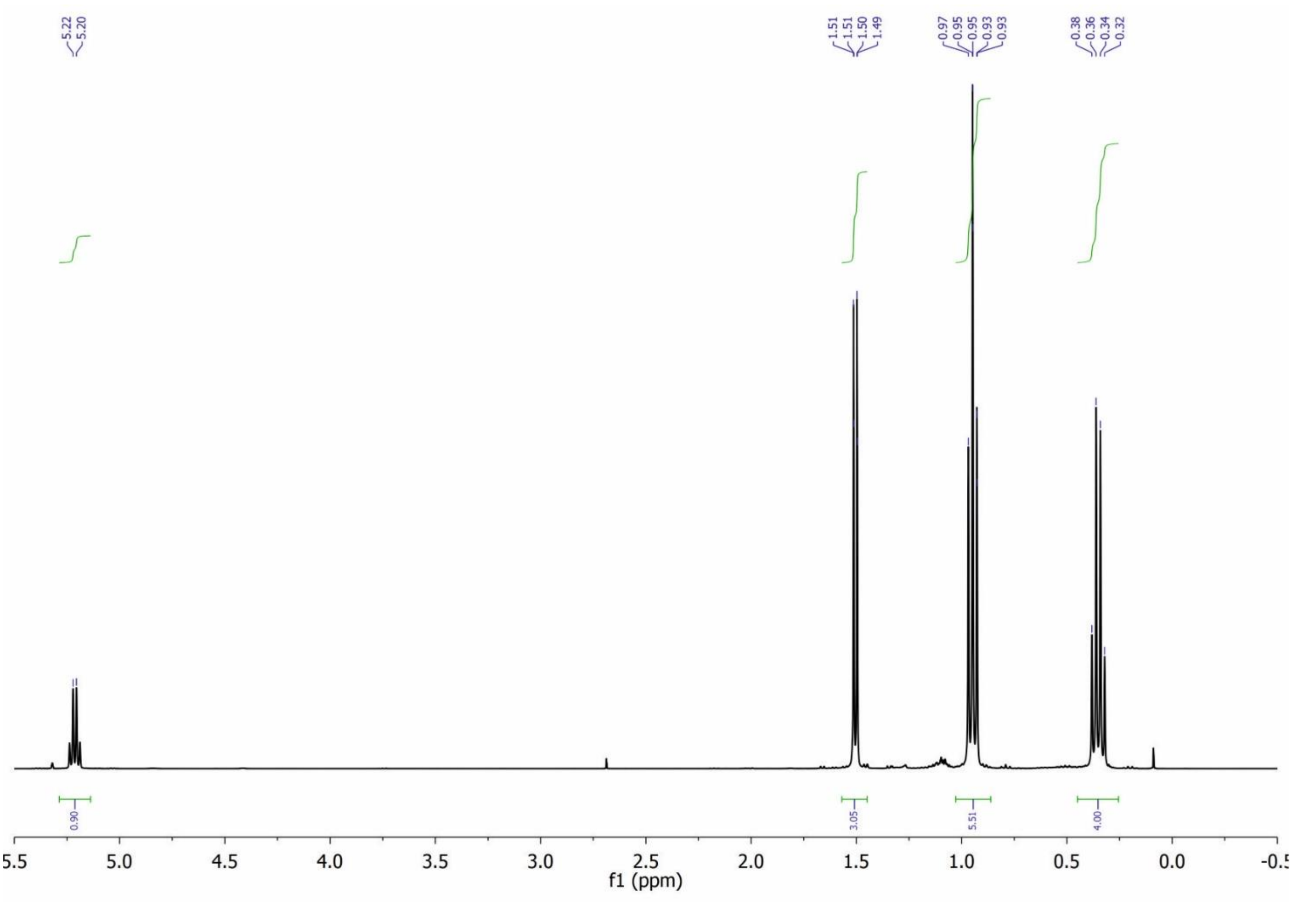

Figure S6. ^{1}H NMR (CD_2Cl_2, 400 MHz) spectra of **3**.

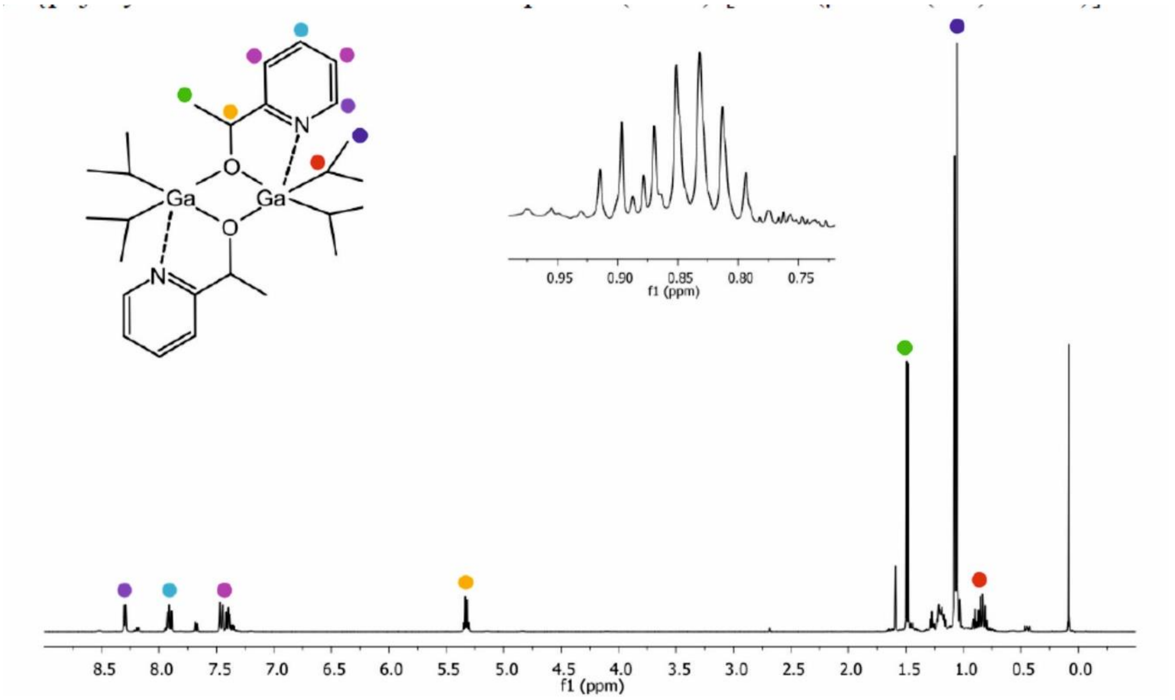

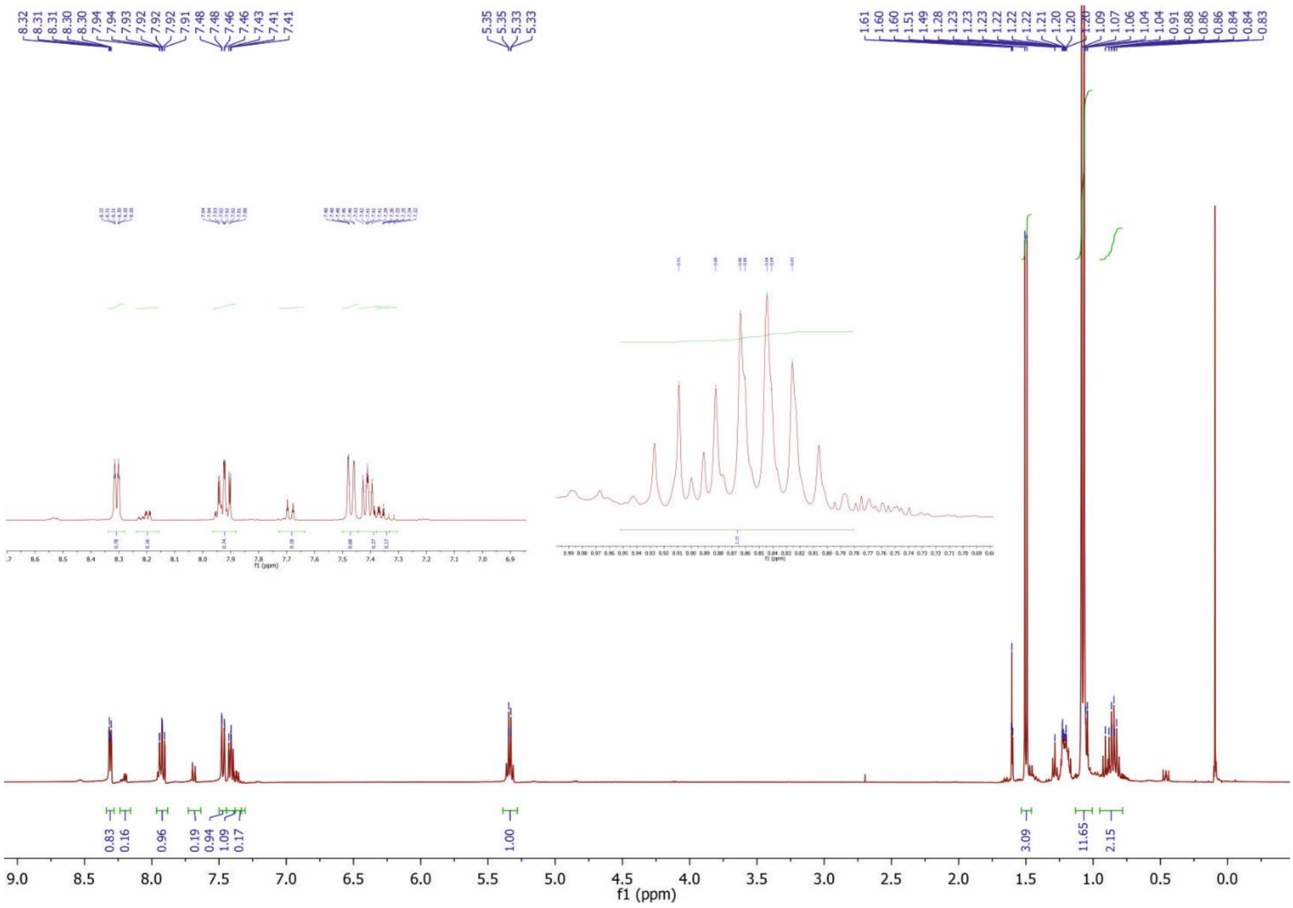

Figure S7. ^{1}H NMR (CD_2Cl_2, 400 MHz) spectra of **4**.

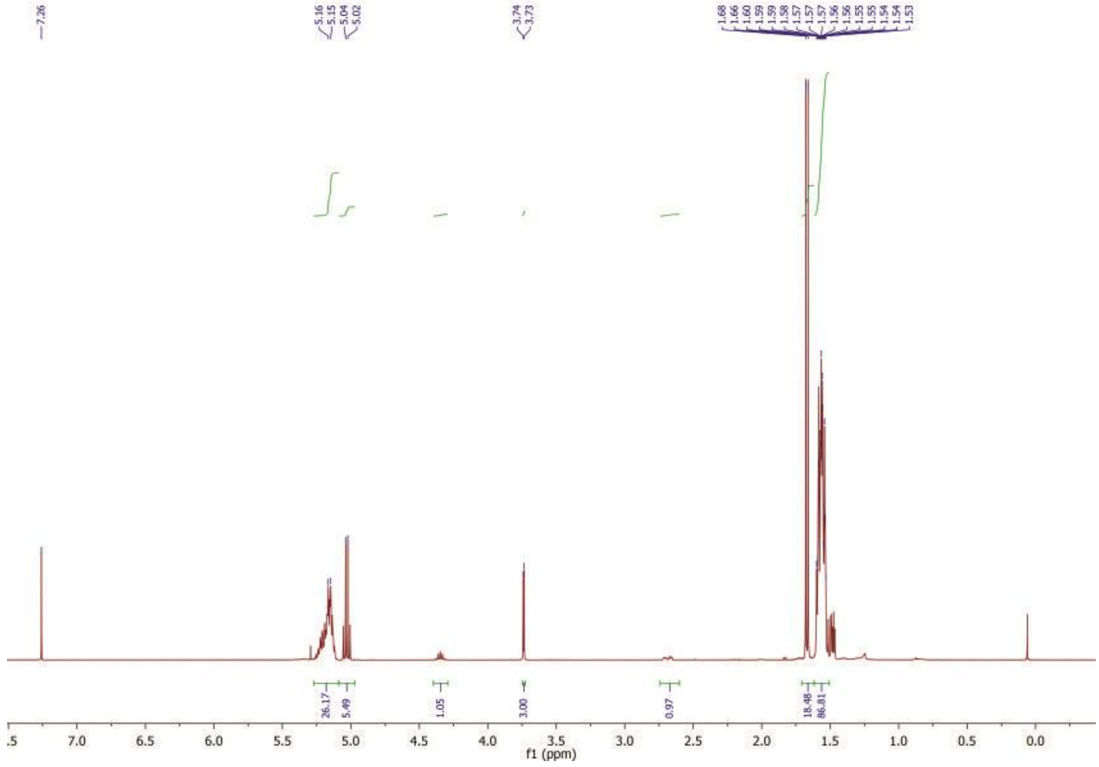

Figure S8. ^{1}H NMR ($CDCl_3$, 400 MHz) Exemplary ^{1}H NMR spectra of PLA obtained with gallium complexes.

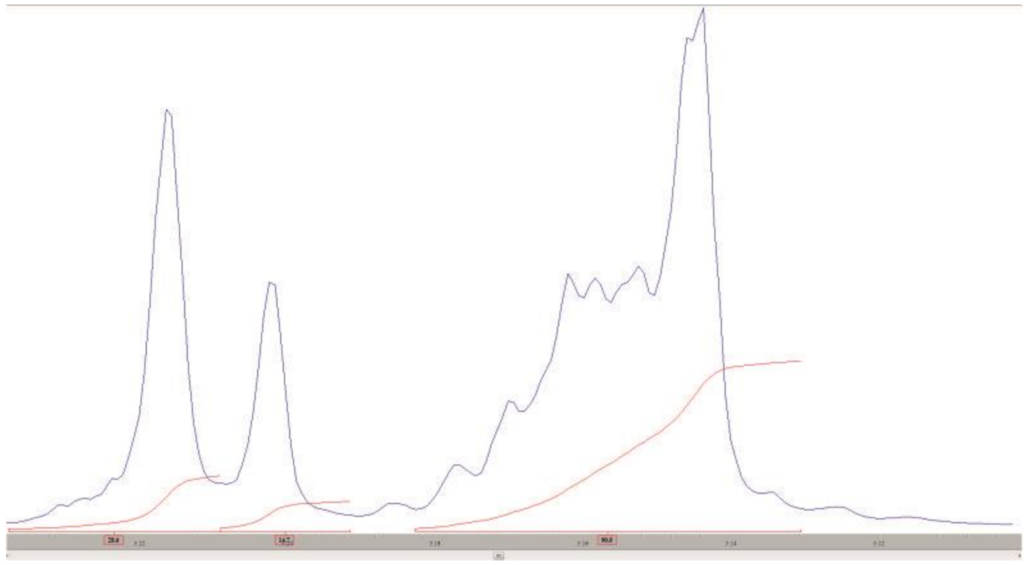

Figure S9. ^1H NMR ($CDCl_3$, 400 MHz) Decoupled ^1H NMR spectra of PLA (methine region) obtained with (*S,S*)-[$Me_2Ga(\mu\text{-}OCH(Me)CO_2Me]_2$/pyridine (1:6) at 40°C.

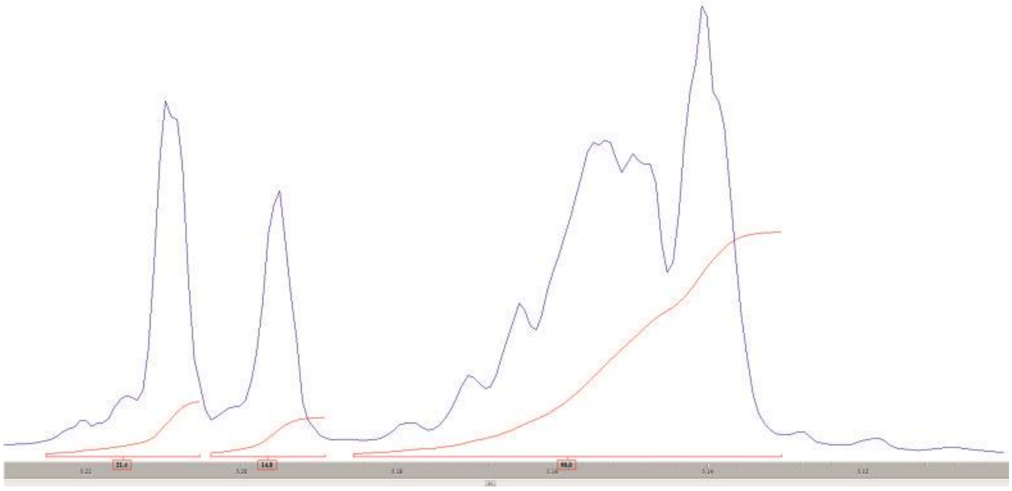

Figure S10. ^1H NMR ($CDCl_3$, 400 MHz) Decoupled ^1H NMR spectra of PLA (methine region) obtained with (*S,S*)-**1**/pyridine (1:6) at 40°C.

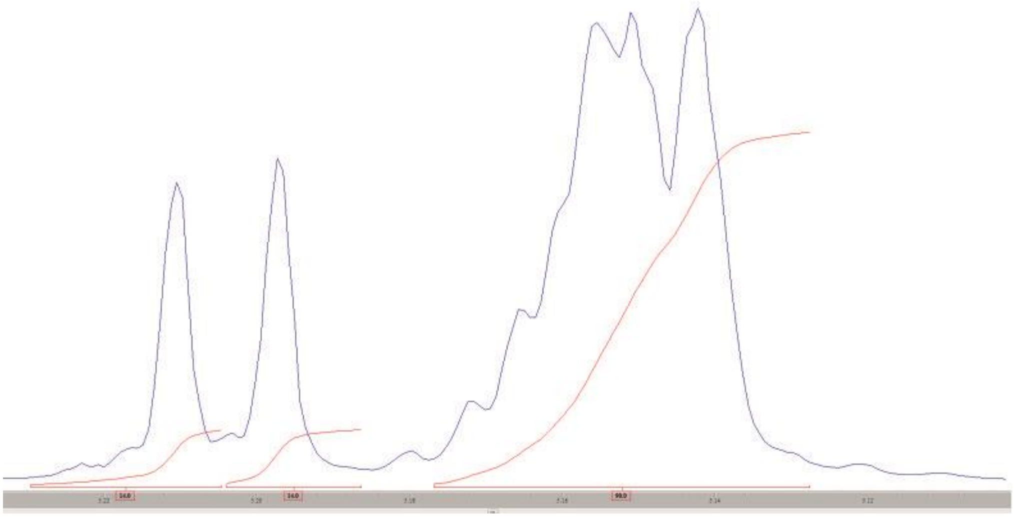

Figure S11. ^{1}H NMR ($CDCl_3$, 400 MHz) Decoupled ^{1}H NMR spectra of PLA (methine region) obtained with (*S,S*)-**1**/pyridine (1:6) at 40°C.

2) FTIR

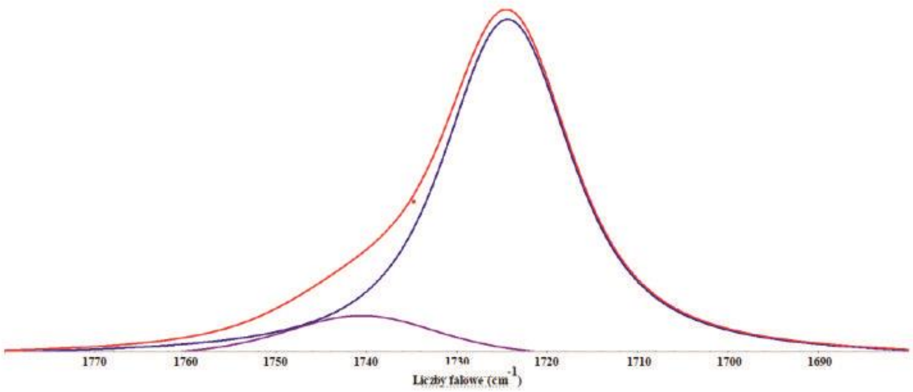

Figure S12. FTIR spectra in the region of C=O band of (*S*,*S*)-**1** (in CH_2Cl_2). The original band is indicated in red. Purple and blue bands represent bands of free (1740 cm^{-1}) and coordinated C=O groups (1724 cm^{-1}), respectively, obtained after deconvolution.

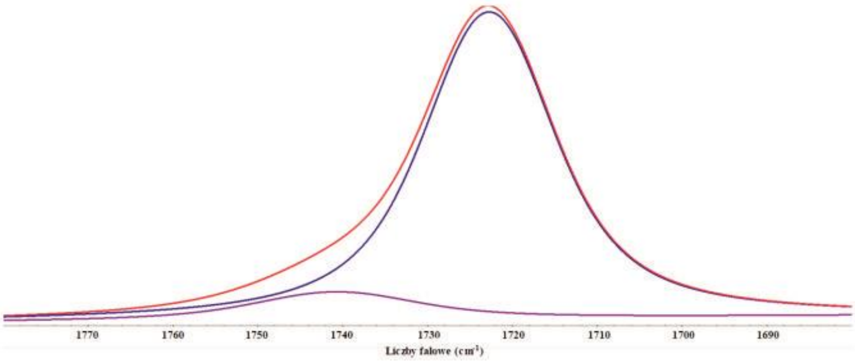

Figure S13. FTIR spectra in the region of C=O band of (*S*,*S*)-**2** (in CH_2Cl_2). The original band is indicated in red. Purple and blue bands represent bands of free (1740 cm^{-1}) and coordinated C=O groups (1724 cm^{-1}), respectively, obtained after deconvolution.

3) MALDI-TOF

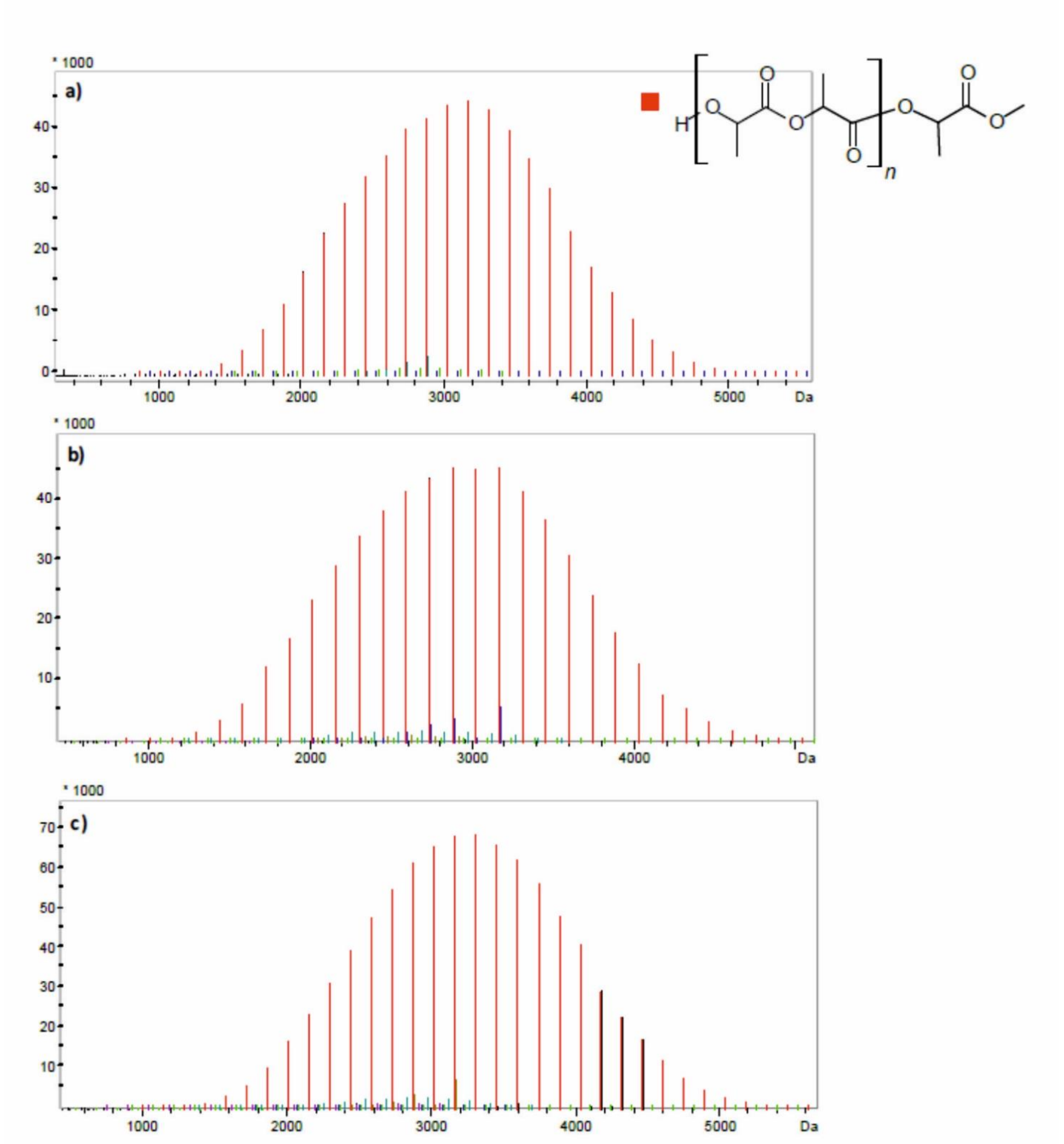

Figure S14. MALDI–TOF spectrum of PLA obtained with: (*S,S*)-[$Me_2Ga(\mu\text{-}OCH(Me)CO_2Me]_2$ / pyridine (1:6) at 40°C (a), (*S,S*)-**1**/pyridine (1:6) at 40°C (b) and (*S,S*)-**2**/pyridine (1:6) at 40°C (c)

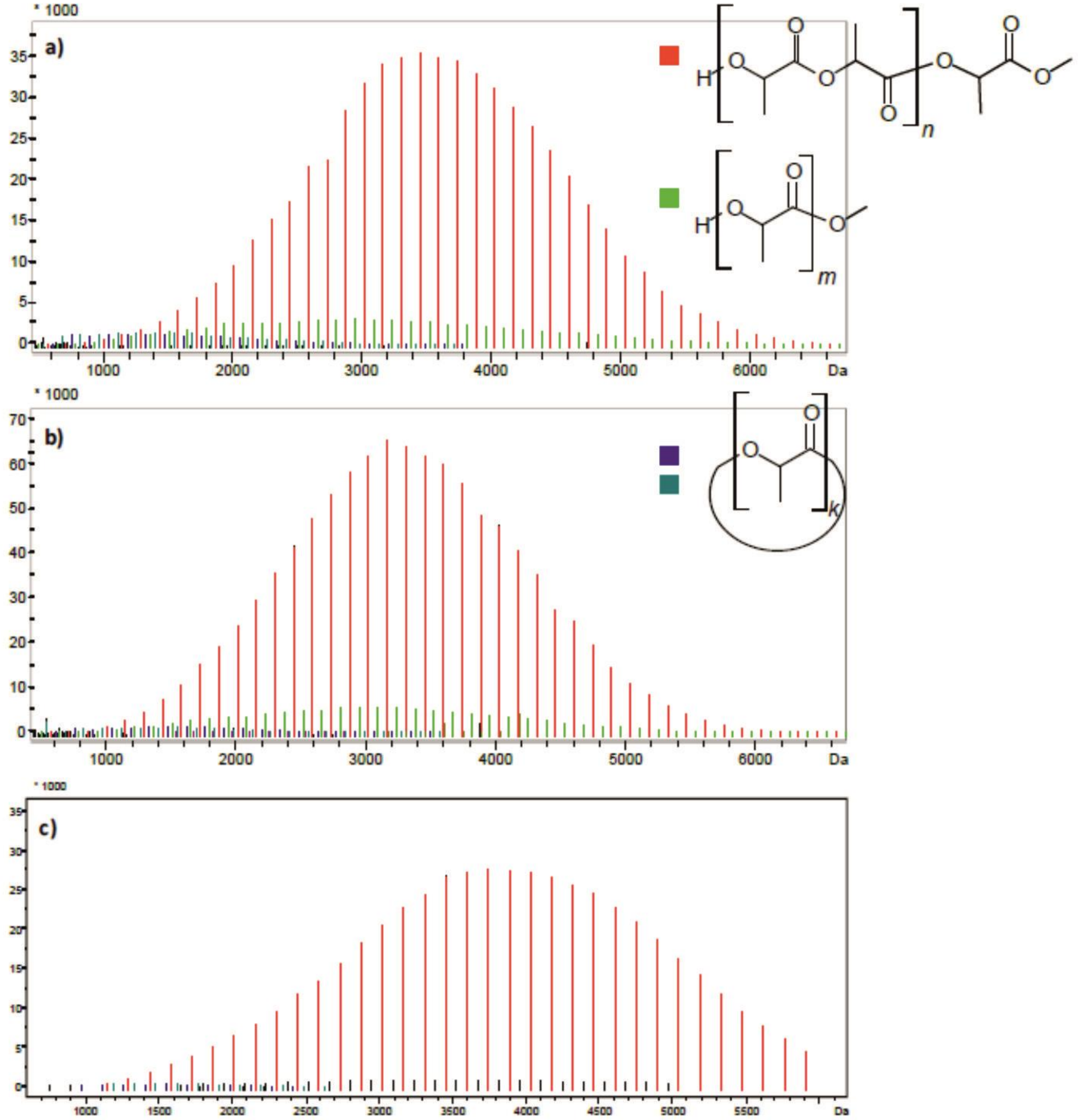

Figure S15. MALDI–TOF spectrum of PLA obtained with: (*S,S*)-[$Me_2Ga(\mu\text{-}OCH(Me)CO_2Me]_2$ at 70°C (a), (*S,S*)-**1** at 70°C (b) and (*S,S*)-**2** at 70°C (c)

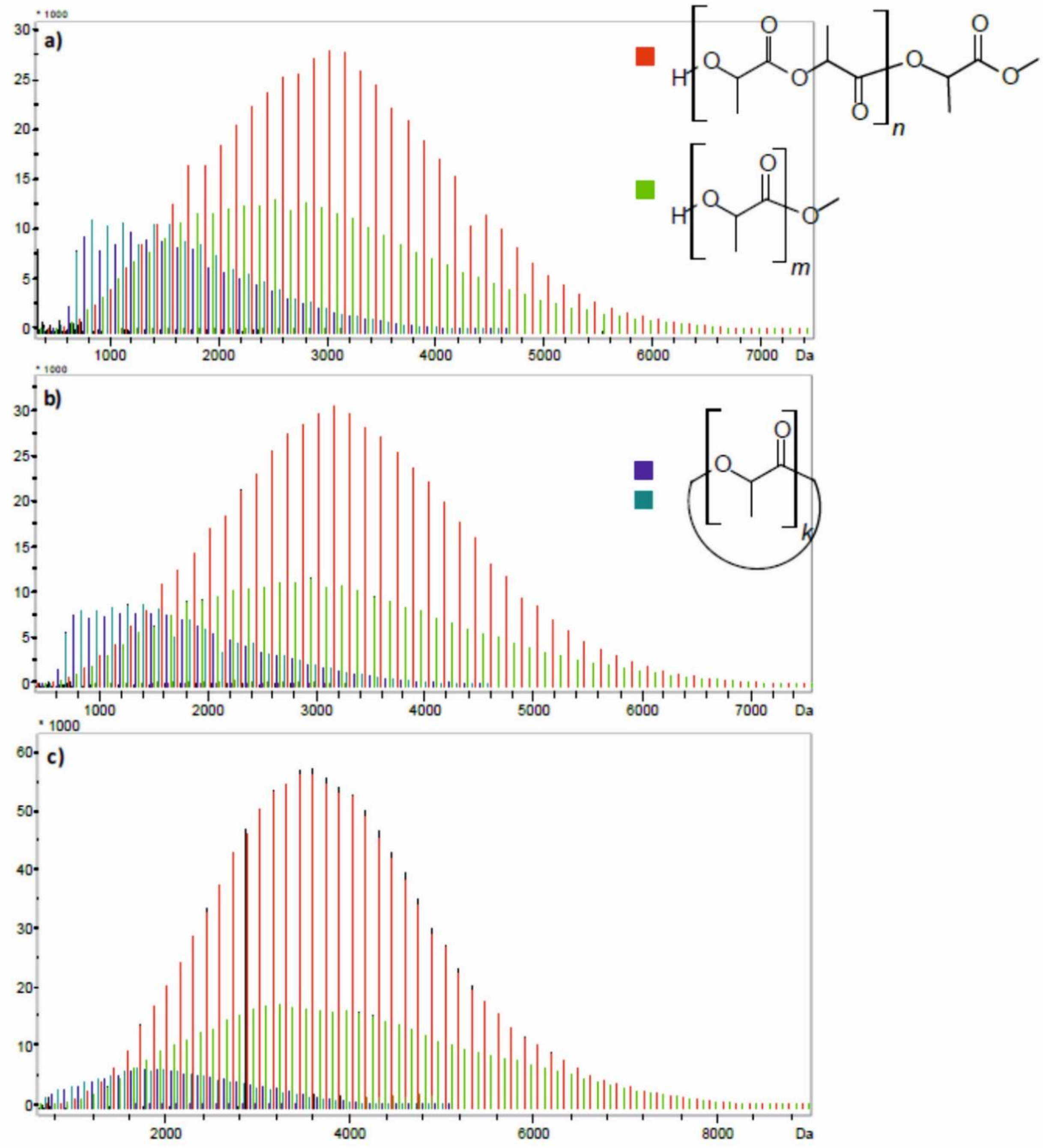

Figure S16. MALDI–TOF spectrum of PLA obtained with: (*S,S*)-[$Me_2Ga(\mu\text{-}OCH(Me)CO_2Me]_2$ / pyridine (1:6) at 70°C (a), (*S,S*)-**1**/pyridine (1:6) at 70°C (b) and (*S,S*)-**2**/pyridine (1:6) at 70°C (c)

4) Crystal data and structure refinement

Table S1 Crystal data and structure refinement details for (R,S)-**2**, (S,S)-**1**, (S,S)-**2**, (R,S)-**3** and (R,S)-**4**.

**Identification code**	(*R*,*S*)-**3**	(*S*,*S*)-**1**	(*R*,*S*)-**2**	(*S*,*S*)-**2**	(*R*,*S*)-**4**
**Formula**	$C_{22}H_{36}Ga_2N_2O_2$	$C_{16}H_{34}Ga_2O_6$	$C_{20}H_{42}Ga_2O_6$	$C_{20}H_{42}Ga_2O_6$	$C_{26}H_{44}Ga_2N_2O_2$
M_x/ g mol^{-1}	499.97	461.87	517.97	517.97	556.07
T/ K	100.0(5)	130.0(5)	130.0(5)	130.0(5)	293.0(1)
λ/ Å	0.71073	0.71073	0.71073	0.71073	0.71073
**Crystal size/ mm**	0.16×0.31×0.34	0.19×0.27×0.33	0.14×0.17×0.26	0.19×0.21×0.30	0.71×0.35×0.21
**Space group**	$P\bar{1}$	$P1$	$P2_1/c$	$P2_1$	$P2_1/n$
**Unit cell dimensions**	a = 8.7984(5) Å<br>b = 8.8724(5) Å<br>c = 8.9171(5) Å<br>α = 82.408(2)°<br>β = 66.358(2)°<br>γ = 67.060(2)°	a = 8.1834(7) Å<br>b = 8.2430(7) Å<br>c = 8.7070(8) Å<br>α = 113.352(3)°<br>β = 90.424(3)°<br>γ = 94.829(3)°	a = 10.5816(5) Å<br>b = 15.3600(5) Å<br>c = 7.9356(3) Å<br>β = 100.808(2)°	a = 7.8664(2) Å<br>b = 15.5512(4) Å<br>c = 10.4906(3) Å<br>β = 100.1397(9)°	*a = 10.0033(3) Å*<br>*b = 12.2497(4) Å*<br>*c = 11.8020(4) Å*<br>*β = 94.142(3)°*
V/ Å^3, Z	587.00(6), 1	536.78(8), 1	1266.92(9), 2	1263.29(6), 2	1442.41(8), 2
D_x/ g cm^{-3}	1.414	1.429	1.358	1.362	1.280
μ/ mm^{-1}	2.313	2.534	2.155	2.161	1.890
F(000)	260	240	544	544	584.0
θ_{min}, θ_{max}	2.49, 30.00°	2.88, 26.49°	2.93, 28.25°	2.94, 25.38	6.652, 55.752°
**Index ranges**	$-12 \leq h \leq 12$<br>$-12 \leq k \leq 12$<br>$-12 \leq l \leq 12$	$-10 \leq h \leq 10$<br>$-10 \leq k \leq 10$<br>$-10 \leq l \leq 10$	$-14 \leq h \leq 14$<br>$-20 \leq k \leq 20$<br>$-10 \leq l \leq 10$	$-9 \leq h \leq 9$<br>$-18 \leq k \leq 18$<br>$0 \leq l \leq 12$	$-13 \leq h \leq 13$,<br>$-16 \leq k \leq 16$,<br>$-15 \leq l \leq 15$
**Reflections collected/ independent**	32854/ 3435 (R_{int} = 0.0215)	15512/ 4348 (R_{int} = 0.0313)	37190/ 3118 (R_{int} = 0.0291)	33428/ 4573 (R_{int} = 0.0203)	20992/ 3440 (R_{int} = 0.0283)
**Completeness**	100.0%	99.9%	99.7%	98.4%	99.8%
**Absorption correction**	Multi-Scan	Multi-Scan	Multi-Scan	Multi-Scan	Multi-Scan
T_{max}, T_{min}	0.708, 0.507	0.645, 0.489	0.752, 0.604	0.684, 0.563	0.486, 0.477
**Refinement method**	Full-matrix LSQ on F^2	Full-matrix LSQ on F^2	Full-matrix LSQ on F^2	Full-matrix LSQ on F^2	Full-matrix LSQ on F^2
**Data / restraints / parameters**	3435 / 54 / 166	4348 / 3 / 225	3118 / 38 / 157	4573 / 1 / 265	3440/39/200
**GOF on** F^2	1.093	1.052	1.126	1.160	1.057
**Final** R **indices**	I>2σ(I)<br>R1 = 0.0156,<br>wR2 = 0.0420<br>all data<br>R1 = 0.0166,<br>wR2 = 0.0426	I>2σ(I)<br>R1 = 0.0186,<br>wR2 = 0.0462<br>all data<br>R1 = 0.0199,<br>wR2 = 0.0466	I>2σ(I)<br>R1 = 0.0208,<br>wR2 = 0.0447<br>all data<br>R1 = 0.0271,<br>wR2 = 0.0485	I>2σ(I)<br>R1 = 0.0209,<br>wR2 = 0.0621<br>all data<br>R1 = 0.0215,<br>wR2 = 0.0668	I>2σ(I)<br>R1 = 0.0295,<br>wR2 = 0.0736<br>all data<br>R1 = 0.0475,<br>wR2 = 0.0827
**Extinction coefficient**	-	-	0.0030(5)	-	-
**Flack parameter**	-	Fit to all intensities: 0.008(14)	-	Fit to all intensities: -0.011(16)	-

		1963 sel. quotients: 0.031(7)		2069 sel. quotients: 0.016(27)	
**$\Delta\rho_{max}$, $\Delta\rho_{min}$**	0.395, -0.249 e·Å^{-3}	0.335, -0.339 e·Å^{-3}	0.323, -0.334 e·Å^{-3}	0.277, -0.252 e·Å^{-3}	
**CCDC number**	2406865	2406867	2406866	2406868	2406869
